# Methodologies for Backbone Macrocyclic Peptide Synthesis Compatible With Screening Technologies

**DOI:** 10.3389/fchem.2020.00447

**Published:** 2020-06-18

**Authors:** Koki Shinbara, Wenyu Liu, Renier Herman Pieter van Neer, Takayuki Katoh, Hiroaki Suga

**Affiliations:** Department of Chemistry, Graduate School of Science, The University of Tokyo, Tokyo, Japan

**Keywords:** non-proteinogenic amino acid, backbone macrocyclic peptide, enzymatic peptide backbone cyclization, peptide library, OBOC screening, SICLOPPS, *in vitro* screening

## Abstract

Backbone macrocyclic structures are often found in diverse bioactive peptides and contribute to greater conformational rigidity, peptidase resistance, and potential membrane permeability compared to their linear counterparts. Therefore, such peptide scaffolds are an attractive platform for drug-discovery endeavors. Recent advances in synthetic methods for backbone macrocyclic peptides have enabled the discovery of novel peptide drug candidates against diverse targets. Here, we overview recent technical advancements in the synthetic methods including 1) enzymatic synthesis, 2) chemical synthesis, 3) split-intein circular ligation of peptides and proteins (SICLOPPS), and 4) *in vitro* translation system combined with genetic code reprogramming. We also discuss screening methodologies compatible with those synthetic methodologies, such as one-beads one-compound (OBOC) screening compatible with the synthetic method 2, cell-based assay compatible with 3, limiting-dilution PCR and mRNA display compatible with 4.

## Introduction

Peptides have the potential to be therapeutic agents in various aspects. Even though they are small in size compared with biological drugs, such as antibodies, they possess unique traits similar to those. Peptides would have a specific and high binding affinity to target proteins of interest and could bind not only to their pocket but also to the relatively flat protein surface (Laraia et al., 2015). However, peptides consisting of ordinary amino acids have challenges to overcome before being an effective therapeutic agent, such as low metabolic stability caused by proteolysis *in vivo*, and poor cell-permeability caused by their larger size, and the aquaphilic property due to multiple hydrogen bonding donors/acceptors in the peptide backbone compared to small molecules. Macrocyclic peptides can potentially overcome these challenges. Their rigid structure contributes to the avoidance of proteolysis (March et al., 1996), and a closed conformation, wherein hydrophobic regions are exposed to the surface but hydrophilic regions are hidden inside of the cyclic structure, increases their cell-membrane permeability (Rezai et al., 2006).

Macrocyclic peptides are classified into three groups by cyclization style: sidechain-to-sidechain, head-to-sidechain, and head-to-tail (backbone) cycles are all found in natural products. Among them, a backbone macrocyclic conformation provides the most conformationally constrained structure due in part to the consecutive, unsaturated amide bonds, which cannot rotate and thus contribute to a more rigid structure. On the other hand, the other methods of cyclization use an amino acid's sidechain. This introduces at least one saturated single bond, expanding their flexibility compared to backbone macrocyclic peptides (Horton et al., 2000).

Despite the fact that there are numerous kinds of backbone macrocyclic peptides originating from naturally occurring peptides and rationally or semi-rationally designed molecules, this chapter focuses on discussing recent technical advancements that allow researchers to discover *de novo* backbone macrocyclic peptides. Some technologies covered in this review have been well-established and successfully applied to discover bioactive molecules in the last decade, but some technologies emerged recently and have thus not yet been fully extended to the discovery of *de novo* macrocyclic peptides. In any case, this review will also cover the discussion of such technologies.

## Enzymatic Peptide Backbone Cyclization

Naturally occurring backbone macrocyclic peptides are generally matured from their linear counterparts by their specific cyclases, some of which have substrate promiscuity, and may be utilized for cyclization of other peptides. These enzymes have been further engineered to cyclize a wide variety of backbone macrocyclic peptides. This section will discuss the characteristics, advantages, and disadvantages of these promiscuous cyclases; asparaginyl endoproteases, sortases, and subtilisin-like variants.

### Asparaginyl Endoprotease-Mediated Backbone Cyclization

**Figure 1:**
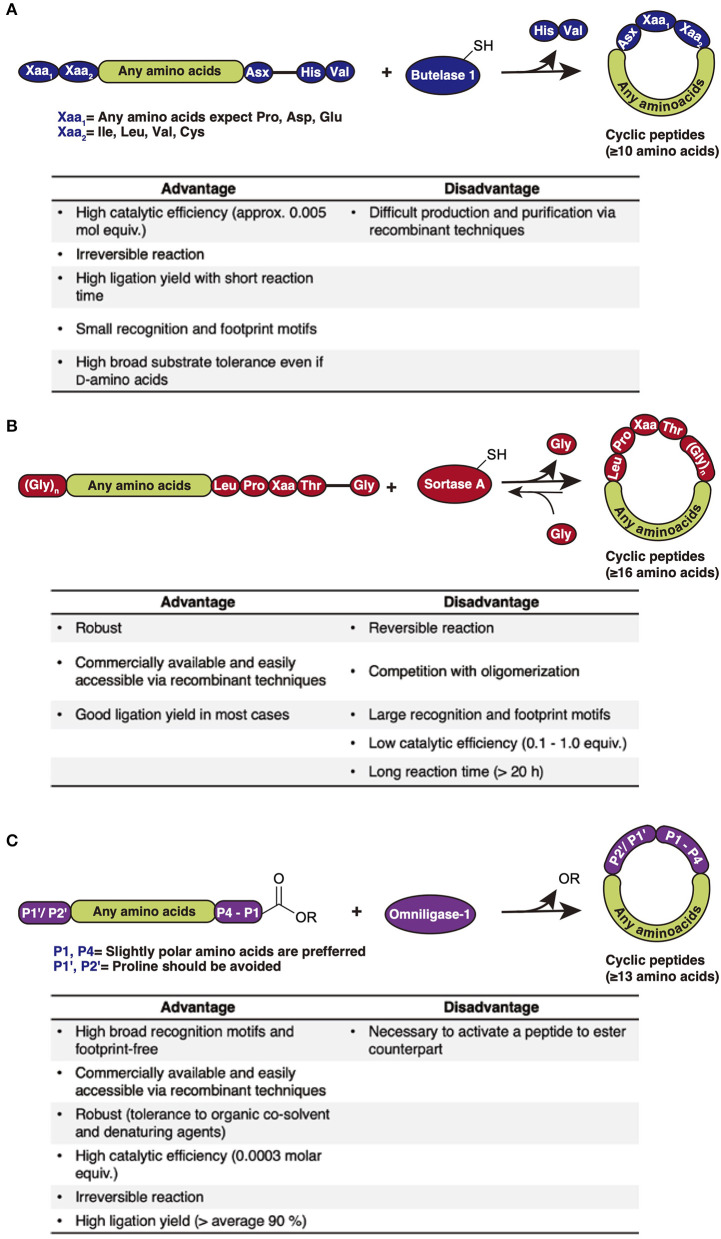
Comparison of cyclization methods and their advantages and disadvantages **(A)** Asparaginyl endoprotease-mediated backbone cyclization (e.g., Buterase-1). Butelase 1 recognizes the C-terminal Asx-His-Val motifs to produce the corresponding backbone cyclic peptide containing the Asx-Xaa_1−_Xaa_2_ sequence and removing the His-Val segment. **(B)** Sortase-mediated backbone cyclization (e.g., Sortase A). Sortase A recognizes the C-terminal Leu-Pro-Xaa-Thr-Gly motifs to produce the corresponding backbone cyclic peptide containing a Leu-Pro-Xaa-Thr-(Gly)_n_ sequence and removing a single Gly. **(C)** Peptiligase variants-mediated backbone cyclization (e.g., Omniligase- 1). Ommiligase-1 recognizes the N-terminal of P1′/P2′ motifs, and the C-terminal P4-P1 ester motifs to produce the corresponding backbone cyclic peptide containing a P1′/P2′, P4-P1 sequence, and removing the ester leaving group.

Asparaginyl endoproteases represent a common protease family in nature. Nevertheless, some of them, such as butelase 1, have the capability to ligate peptide bonds with certain recognition motives. Butelase 1, which is isolated from the tropical plant *Clitoria ternatea*, is involved in the biosynthesis of cyclotides (Nguyen et al., 2014). Butelase 1 recognizes a C-terminal Asx-His-Val motif (Asx = Asp or Asn) and cleaves the His-Val segment from the peptide to form a thioester acyl-enzyme intermediate (Figure 1A). The backbone macrocyclic peptide, containing the remaining Asx, is then produced through nucleophilic attack by the peptide's N-terminal amino group. Although the acyl-enzyme intermediate could be attacked by the leaving His-Val segment, the cyclization is irreversible. Although the N-terminal Xaa_1_ and Xaa_2_ amino acids are little limited (Xaa_1_ = any amino acids except Pro, Asp, and Glu, Xaa_2_ = Ile, Leu, Val, or Cys), butelase 1 has an impressive catalytic efficiency, requiring only 0.005 molar equivalents in cyclization reactions.

Utilizing butelase 1, several backbone macrocyclic peptides composed of proteinogenic amino acids (PAAs) such as katala B1 were successfully cyclized within minutes with high efficiency (>95%) (Nguyen et al., 2015). A peptide containing almost all d-amino acids could also be processed (Nguyen et al., 2016).

The main disadvantage of butelase 1 is the difficulty of its production. Thus far, recombinant production of butelase 1 has not been achieved, and it can only be acquired through purification from plant materials (Nuijens et al., 2019).

### Sortase-Mediated Backbone Cyclization

Sortase A, isolated from *Gram-positive Staphylococcus aureus*, is a transpeptidase that catalyzes the covalent ligation of bacterial surface proteins to the bacterial cell wall (Mazmanian et al., 1999). Sortase A recognizes a C-terminal Leu-Pro-Xaa-Thr-Gly motif and cleaves the terminal Gly to subsequently link the threonyl carboxylate to the pentaglycine of a peptidoglycan unit. Sortase A has been employed for both peptide-protein ligation and backbone cyclization. Sortase-mediated backbone cyclization requires the precursor peptide to contain the C-terminal Leu-Pro-Xaa-Thr-Gly motif and an N-terminal (oligo) Gly (Figure 1B) (Huang et al., 2003; Mao et al., 2004). Using sortase A, natural bioactive cyclotides such as katala B1, a sequence grafted cyclic peptide derived from the sunflower trypsin inhibitor SFT-1, and a macrocyclized version of the 38-mer salivary peptide histatin 1 were successfully synthesized (Bolscher et al., 2011; Jia et al., 2014; Zhang et al., 2015).

Sortase 1 can be produced with recombinant techniques owing to its robustness. It is also commercially available and achieves high conversion yield of products. However, there are several limitations to this approach. First, sortase A-cyclized backbone cyclic peptides have a large >5 residue sized footprint in the final sequence. Second, the low catalytic efficiency of sortase A (a 0.1–1.0 molar ratio of enzyme to substrate is required) results in long reaction times, often of over 20 h. Third, due to the reversible reaction derived from transpeptidase activity, product cleavage may occur. These limitations hindered the use of sortase A for peptides shorter than 16 mer, predominantly yielding oligomerized peptides rather than cyclized ones (Wu et al., 2011).

### Subtilisin-Like Variant-Mediated Backbone Cyclization

Subtiligase is a peptide cyclase derived from a subtilisin protease, substilisin BPN', from *Bacillus amyloliquefaciens* (Braisted et al., 1997). In subtiligase, a Ser in the active site of substilisin BPN' was substituted with Cys to promote acylation over the hydrolysis, and its neighboring Pro was substituted with Ala to reduce steric crowding. The substituted Cys provokes a nucleophilic attack from a substrate acyl donor to form a thioester-acyl enzyme intermediate, which is subsequently attacked by an N-terminal amino group to produce a ligation product. However, the yield of this reaction is not high, with it giving more or less 60% due to hydrolytic side reactions. Recently, a novel and robust subtilisin-based variant termed peptiligase was developed by introducing the same Ser-to-Cys and Pro-to-Ala mutations into a calcium-independent and stable variant of substilisin BPN' (Toplak et al., 2016). Peptiligase is easily accessible through recombinant expression from *Bacillus subtilis*. Furthermore, an improved peptiligase variant, omniligase-1, was developed to broaden the substrate scope. Omniligase-1 catalyzes the peptide ligation between a C-terminal ester [preferably carboxyamidomethyl (Cam) ester] and an N-terminal amino group with significantly reduced rates of ester hydrolysis (Figure 1C). Although it recognizes the C-terminal of P1′/P2′ and the N-terminal of P1-P4 sequences, omniligase-1's broadened substrate scope (slightly polar amino acids are preferred at the P1, P4 position, and proline should be avoided at the P1′, P2′ position) allows it to be used for footprint-free backbone cyclization, enabling a ligation reaction of unprotected peptides at ambient temperature with high yield in a short time (up to 90% in <1 h) in aqueous media with neutral to slightly basic pH. Additionally, organic co-solvent media (e.g., up to 50 vol% of DMF and DMSO) and denaturing agents (e.g., 2M urea or guanidium chloride) are tolerated in the ligation reaction, allowing poorly soluble peptides to be cyclized (Toplak et al., 2016). Omiligase-1 has a high catalytic efficiency (<0.0003 molar equivalents of enzyme required) and is commercially available. Therefore, the cyclization reaction is easily scaled up to produce quantitative yields (Nuijens et al., 2016). Omniligase-1 has been successfully utilized for the backbone cyclization and oxidative folding of the disulfide-rich peptides, MCoTI-II, RTD-1, katala B1, and their variants in a one-pot reaction (Schmidt et al., 2019). Furthermore, several backbone macrocyclic peptides containing d-amino acids, isopeptide bonds, and non-peptidic moieties such as polyethyl glycol could be efficiently cyclized (Schmidt et al., 2017). The only major limitation of omniligase-1 is the requirement for an ester moiety at the substrate's C-terminus.

Asparaginyl endoproteases, sortases, and subtilisin-like enzymes are very powerful tools for the synthesis of a wide variety of backbone macrocyclic peptides. Although several model cyclic peptides can be synthesized, there are as of yet few reports about the construction or screening of cyclic peptide libraries using these tools (Nguyen et al., 2016). However, these enzymatic reactions will be coupled with library construction and screening methodologies, which we will mention below, and will accelerate the discovery of bioactive backbone macrocyclic peptides.

## Chemically Synthesized Backbone Macrocyclic Peptides and Their Applications

### Solid-Phase Synthesis of Backbone Macrocyclic Peptides

**Figure 2:**
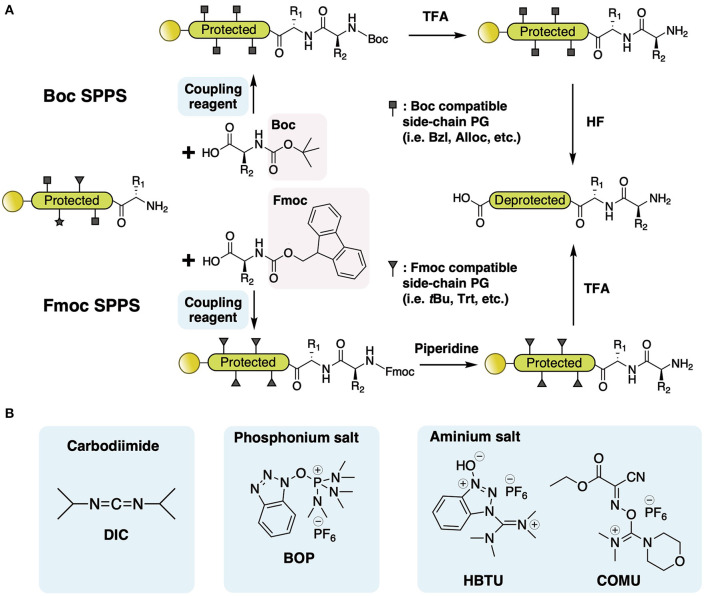
**(A)** Comparison of Boc and Fmoc SPPS. Fmoc/*t*Bu SPPS employs removal of the amine-protecting Fmoc group in an alkaline condition (normally 20% piperidine) and eventual cleavage of orthogonal side-chain protecting groups, including *t*Bu and the support linker, by high-percentage TFA, whereas Boc/bzl SPPS requires harsher reaction conditions, using 50% TFA for amine-protecting Boc removal and 90% HF for protecting groups and support linker cleavage. **(B)** Examples of three classes of coupling reagent, carbodiimides, phosphonium salts, and aminium salts.

The past century has witnessed a drastic development of peptide synthesis methodologies since early chemical synthesis of the dipeptide glycylglycine (GlyGly) was achieved in solution phase by Fischer in the late nineteenth century (Fischer and Fourneau, 1901). Advances in protecting groups (amino- and side chain-protection) and coupling reagents have been made since to allow consecutive elongation of amino acid residues and improve coupling efficiency. A great evolution has occurred in peptide synthesis since Merrifield pioneered the idea of solid-phase peptide synthesis (SPPS), and it has become the main synthetic approach for peptides and small proteins smaller than 100 residues in current laboratories (Merrifield, 1963; Palomo, 2014). Compared with peptide synthesis in solution-phase, SPPS provides a simple, fast, and efficient route, during which the whole peptide is assembled in a single reaction vessel and no excessive purification is required for reaction intermediates, therefore avoiding loss of yield. The basic concept of SPPS includes repetitive coupling and the deprotection of N-terminal and side-chain-protected amino acids to an insoluble polymeric support, followed by an eventual deprotection of all side-chain protecting groups and cleavage from the support. Modern SPPS synthesis utilizes two standard strategies based on the N-terminal protection, fluorenylmethyloxycarbonyl/*tert*-butyl (Fmoc/*t*Bu) and *tert*-butyloxycarbonyl/benzyl (Boc/bzl) (Figure 2A) (Isidro-Llobet et al., 2009). Fmoc/*t*Bu SPPS employs the removal of the amine-protecting Fmoc group in an alkaline condition (normally 20% piperidine) and eventual cleavage of orthogonal side-chain protecting groups including *t*Bu and the support linker by high-percentage TFA, whereas Boc/bzl SPPS requires harsher reaction conditions, using 50% TFA for amine-protecting Boc removal and 90% HF for protecting groups and support linker cleavage. Besides protecting groups, various coupling methods have been developed to further improve reaction yield and reduce potential racemization, including the addition of coupling reagents to facilitate carboxylic acid activation. Coupling reagents are of three major classes, carbodiimides, phosphonium, and aminium salts (Figure 2B), varying in their chemical conformations and electrophilic properties. The coupling reagents drastically boost amide bond formation speed and are therefore broadly used in current laboratory work. Nowadays, fully automized peptide synthesizers allow the repetitive steps to be executed more reliably and conveniently by eliminating human errors and attention, achieving parallel synthesis and application of abundant reaction conditions (i.e., coupling reagents, reaction temperature, and time, etc.).

**Figure 3:**
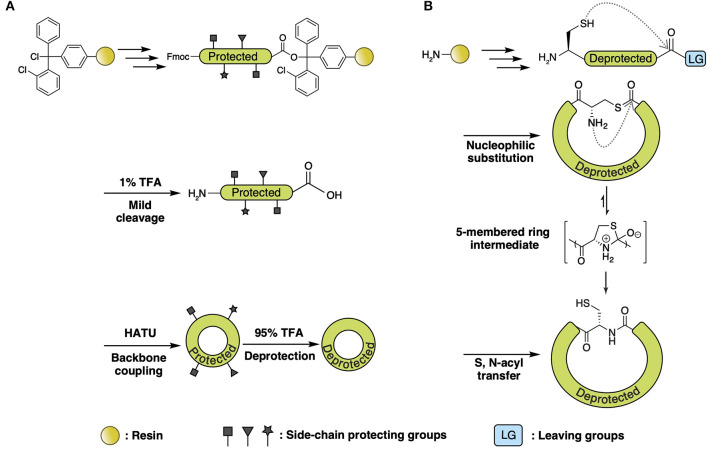
**(A)** Direct head-to-tail coupling of side-chain-protected peptide. Peptide is assembled on chlorotrityl resin followed by 1% TFA cleavage. Backbone cyclization of the side-chain-protected peptide is performed in solution with uronium salt (HATU) and then deprotected with high-percentage TFA. **(B)** Backbone cyclization via S-to-N-acyl transfer. Generally, the peptide is deprotected and cleaved from resin using high-percentage TFA, with the C-terminal acyl connected to a good leaving group (i.e., thioester and azide, etc.) generated from either a chemical linker or additional treatment (discussed in the text). Head-to-tail cyclization is achieved with automatic nucleophilic substitution of C-terminal acyl derivatives with free thiol on N-terminal Cys, leading to the formation of a thioester intermediate. The intermediate subsequently rearranges via intramolecular S-to-N acyl transfer, forming a five-membered transition state that then forms into the backbone amide bond.

With SPPS and further chemical modifications, various side-chain-involved cyclization tactics through lactam bridges (Taylor, 2002), disulfide bonds (Postma and Albericio, 2014), and thiolactone linkage (Van Lysebetten et al., 2018) have been reported in the past two decades. In contrast, SPPS of amide bond-joined backbone macrocyclic peptides proved to be difficult due to the limitation of acyl-derivatives that can react with the solid phase and C-terminal residue. There are two major synthetic strategies for backbone macrocyclic peptides using SPPS and extended chemical methods: direct head-to-tail coupling of side-chain-protected peptides (Figure 3A) and backbone cyclization via S-to-N acyl transfer (Figure 3B).

A direct head-to-tail coupling method is generally used for Fmoc-based SPPS of cyclotides (Cheneval et al., 2014). This approach involves regular SPPS peptide synthesis on chlorotrityl resin followed by mild cleavage (1% TFA). Backbone cyclization of the side-chain-protected peptide is performed in solution with uronium salt, after which, the peptides are deprotected with high-percentage TFA, followed by oxidative disulfide formation. Since the N-terminal amine and C-terminal carboxylic acid of the peptide are both free after mild cleavage, head-to-tail ligation can be achieved by a coupling reagent such as DCC/HOBt in alkaline conditions. This cyclization method is straightforward and accessible due to its compatibility with Fmoc chemistry and the universality of terminal residue choice. Direct head-to-tail coupling was adopted in the synthesis of the cyclotide family for the development of cell-penetrating and inhibitor peptides (Henriques et al., 2015; Huang et al., 2015; D'Souza et al., 2016).

Backbone cyclization via S-to-N acyl transfer is adaptable for both Fmoc- and Boc-based SPPS. The principle chemistry employed for this ligation is nucleophilic substitution of C-terminal acyl derivatives with the unprotected thiol on N-terminal Cys, leading to the formation of a thioester intermediate. This intermediate subsequently rearranges via intramolecular S-to-N acyl transfer, forming a backbone amide bond after passing through a five-membered heterocyclic transition state (Burke et al., 2017).

**Figure 4:**
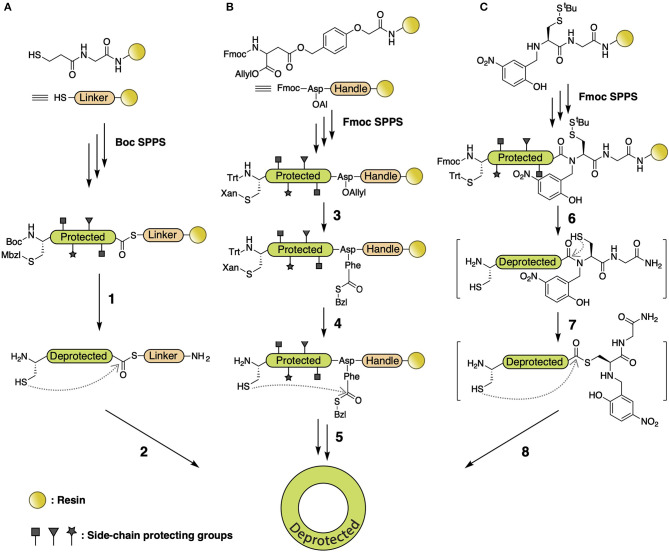
**(A)** NCL of Kalata B1 using -SCH_2_CH_2_CO- as leaving group. A -SCH_2_CH_2_CO- linker is connected to PAM resin, followed by Boc SPPS to elongate the peptide. Cleavage and deprotection are performed with HF (1), and backbone autoligation is reached at pH 7.5 (2). **(B)** NCL of depsipeptide using -SBzl as leaving group. An α-carboxyl-protected Fmoc-Asp-OAllyl is attached to resin with side-chain carboxyl, followed by peptide elongation and deprotection of the allyl group by Pd(Ph_3_)_4_, allowing coupling of Phe-SBzl with HATU/TIEA (3). N-terminal Cys and C-terminal Phe is deprotected with 1% TFA (4) and cyclized with 6 M Gdn.Cl at pH 7.5, followed by deprotection with 90% TFA (5). **(C)** NCL of cDRPs using N-Hnb-Cys crypto-thioester as leaving group. An N-Hnb-Cys is assembled on resin before peptide assembly by Fmoc SPPS, followed by side-chain deprotection with 95% TFA (6). S-^t^Bu is deprotected with 50 mM tris(2-carboxyethyl)phosphine (TCEP) and N-to-S acyl transfer (7) followed by intramolecular NCL induced in 100 mM 4-mercaptophenylacetic acid (MPAA) at 37°C under argon atmosphere (8).

Among all cyclization methods that utilize S-to-N acyl transfer, native chemical ligation (NCL) (Agouridas et al., 2019) is the most broadly used synthetic approach, employing a C-terminal thioester as the acyl donor. Various NCL branches have been established based on thioester generation. In the first chemical synthesis of cyclotide Kalata B1 (Daly et al., 1999; Tam et al., 1999), a -SCH_2_CH_2_CO- linker was connected to PAM resin, followed by automated Boc-standard SPPS to elongate the peptide. Cleavage and deprotection present a H_2_N-Cys-peptide-thioester-linker-Gly-OH intermediate, which is then backbone cyclized via thiol-thioester exchange and S-to-N acyl transfer (Figure 4A) (Clark et al., 2006; Ji et al., 2013; Wang et al., 2014). A subsequent study utilized α-carboxyl-protected Fmoc-Asp-OAl to attach to the resin with a side-chain carboxyl, followed by peptide elongation and deprotection of the α-carboxyl on Asp-OAl, allowing coupling of a Phe-SBzl to the α-carboxyl. Thus, the generated thioester intermediate leads to a backbone macrocyclic decapeptide (Figure 4B) (Tulla-Puche and Barany, 2004). Recent studies used N-to-S acyl transfer to form a thioester on the C-terminus by incorporating N-Hnb-Cys on the resin, followed by intramolecular NCL to yield the desired macrocyclic product (Figure 4C) (Lelièvre et al., 2016; Terrier et al., 2017).

**Figure 5:**
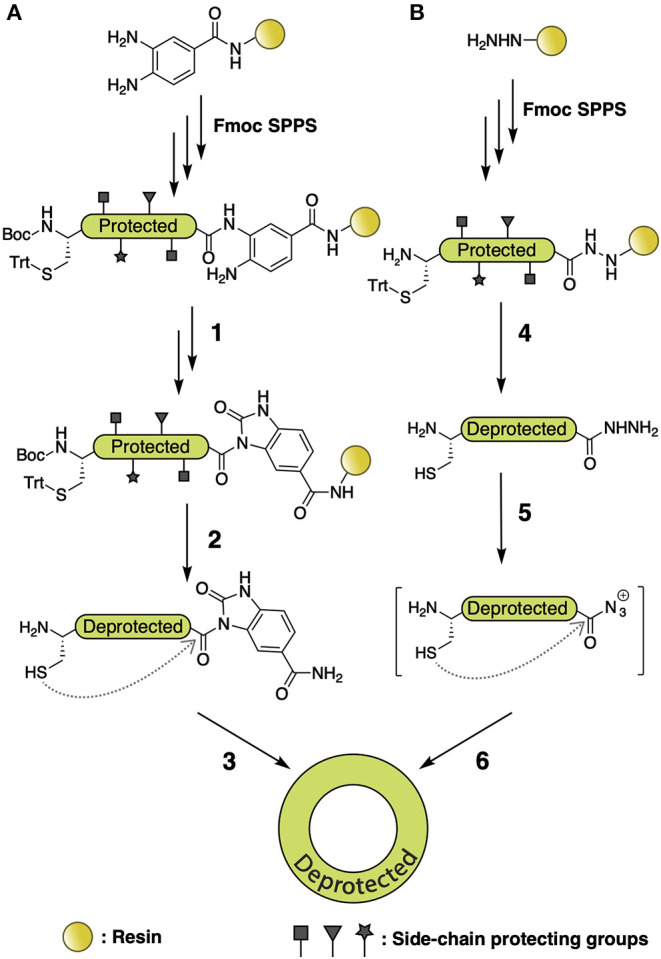
**(A)** Fmoc *N-*acylurea strategy for backbone macrocyclic peptide synthesis. After peptide assembly, C-terminus is activated to an *N-*peptidyl-urea by addition of 4-nitrophenyl chloroformate (1), followed by cleavage and purification (2). The *N-*peptidyl-urea is subsequently cyclized by thiolysis and S-to-N acyl transfer (3). **(B)** Hydrazide methodology for backbone macrocyclic peptide synthesis. Fmoc SPPS is performed using hydrazine-Trt(2-Cl) resin, followed by cleavage and generation of a C-terminal peptide hydrazide (4), which is afterward activated with NaNO_2_ to provide a C-terminal azide (5). That can be used for head-to-tail thiol exchange and S-to-N acyl transfer (6).

Besides NCL, there are numerous methods for backbone cyclization via S-to-N acyl transfer, including the Fmoc *N-*acylurea strategy (Figure 5A) (Blanco-Canosa et al., 2015) and the hydrazide methodology (Figure 5B) (Zheng et al., 2012). The aforementioned Fmoc *N-*acylurea strategy is commonly performed on 3,4-diaminobenzoic acid Rink amide resin. After peptide assembly, the C-terminus is activated using *N-*peptidyl-urea as the acyl donor, followed by cleavage and purification. The *N-*peptidyl-urea is subsequently cyclized by thiolysis and converted into a peptide bond through S-to-N acyl transfer. The hydrazide methodology utilizes hydrazine-Trt(2-Cl) resin to perform peptide assembly, which, after cleavage, generates a C-terminal peptide hydrazide that is then activated with NaNO_2_ to yield an azide for thiol-exchange

We summarized the applications of SPPS methods for backbone macrocyclic peptides (Table 1). Overall, SPPS with extended chemical methods has facilitated a wide range of successful macrocyclic peptide syntheses and has been pivotal for functional studies and pharmaceutical applications. It has enabled facile sequence modification for macrocyclic peptide-based drug design and the production of synthetic substrates to study their structural conformation. We will now describe examples of these and other applications of macrocyclic peptides to probe biological processes and pathways.

**Table 1:** Examples of backbone macrocyclic peptides synthesized with various methods.

**Synthetic method**	**Leaving group**	**Peptide (analogs)**	**Application**	**References**
Direct coupling	None	KB1	Cell-penetrating ability	Henriques et al., 2015
	None	MCoTI-II	Inhibition against BCR-ABL, antagonist of SET	Huang et al., 2015; D'Souza et al., 2016
S-to-N acyl transfer (NCL)	-SCH_2_CH_2_CO- linker	KB1	Structural study of cyclotide plasticity, treatment of multiple sclerosis	Clark et al., 2006; Wang et al., 2014
	-SCH_2_CH_2_CO- linker	MCoTI-II	p53 tumor suppressor	Ji et al., 2013
	-SBzl	Cyclic decamer	Unspecified	Tulla-Puche and Barany, 2004
	N-Hnb-Cys	KB1, cO2, CterM, RTD-1, SFTI-1	Unspecified	Terrier et al., 2017
	N-Hnb-Cys	*Arabidopsis halleri* PDF1.1b	Regulation of zinc tolerance in plants	Lelièvre et al., 2016
S-to-N acyl transfer (non-NCL)	N-acylurea	KB1, MCoTI-II	Unspecified	Blanco-Canosa et al., 2015
	Hydrazide	KB1, cO2, MCoTI-II	Unspecified	Zheng et al., 2012

### One-Bead-One-Compound Method for Backbone Macrocyclic Peptide Screening

Recent pharmacological research has discovered numerous bioactive molecules with various screening techniques that determine functional hits (i.e., small molecules, peptides, and antibodies) from a large candidate pool. As promising drug templates, backbone macrocyclic peptides have been obtained by increasing numbers of accessible and efficient synthetic strategies, facilitating various ring sizes, implementing non-proteinogenic amino acids (NAAs), and enabling diverse conformations and functionalities. However, most of these methods require accurate incorporation of certain amino acids at specific positions, thus leading to repetitive work to make a library containing diverse molecules. To solve this problem, the construction of combinatorial macrocyclic peptide libraries by a split-and-pool synthetic methodology was established (Lam et al., 1991).

**Figure 6:**
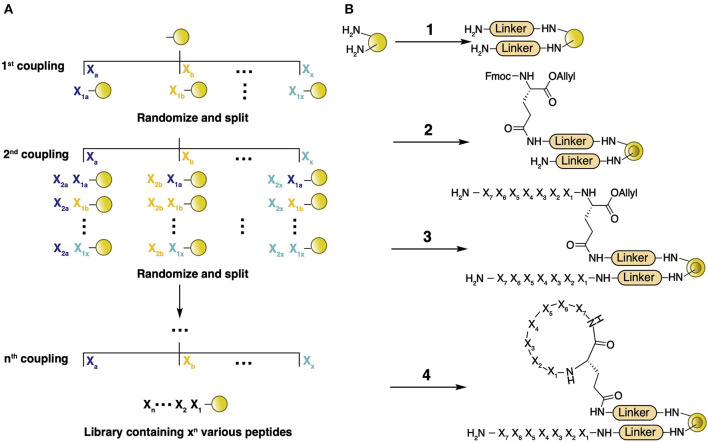
**(A)** General concept of library construction by the split-and-pool method. TentaGel microbeads are prepared and split into X fractions, followed by coupling of X proteinogenic and nonproteinogenic residues onto each fraction separately. The beads are combined and mixed well, followed by splitting again into X fractions. The process of coupling, combining, and splitting is repeated for n cycles, providing a library containing a theoretical X^n^ different molecules. **(B)** Backbone macrocyclic library preparation. TentaGel microbeads are prepared by the addition of a few amino acids (i.e., β-Ala and Met, etc.) as the linker (1), followed by soaking in water and addition of 0.5 equiv. of *N*^α^-Fmoc-Glu(δ-NHS)-O-CH_2_CH=CH_2_ in Et_2_O/CH_2_Cl_2_ (2). Peptide assembly is conducted with Fmoc SPPS (3). Deprotection of the allyl group by Ph(PPh_3_)_4_ is conducted, followed by PyBOP/HOBt head-to-tail coupling of the protected peptide (4).

Generally, the process of screening using a combinatorial macrocyclic peptide library includes library construction on polystyrene beads, screening against a selection target, and post-screening hit sequence identification (Qian et al., 2015). In this methodology, the library is assembled on TentaGel microbeads via an appropriate linker (normally includes β-Ala to enhance protein binding, Met to facilitate peptide release with CNBr, and Arg to provide a positive charge for MS analysis). The linker-modified beads are soaked in water and, through the addition of 0.5 equiv. of *N*^α^-Fmoc-Glu(δ-NHS)-O-CH_2_CH=CH_2_, each bead is spatially generated into outer and inner layers. Peptide assembly is conducted on both layers using a general split-and-pool method (Figure 6A) (Lam et al., 1991), followed by deprotection of the allyl group and head-to-tail coupling of the peptide on only the outer layer (Figure 6B). This process generates a diverse macrocyclic peptide library with a theoretical molecular abundance of 10^7^ species and utilizes the linear peptide as an encoding tag for later hit identification. Since a single bead bears both the cyclic and linear version of the same peptide sequence, the library is termed a one-bead-one-compound (OBOC) library. High-throughput screening of an OBOC library against a specific protein target involves a multistage screening protocol composed of magnetic bead sorting, an enzyme-linked assay, and fluorescence-based screening, which quickly isolates the positive hits from millions of beads (Joo et al., 2006). Since the number of initial hits is still too large to be individually synthesized, the binding affinity of each hit is tested separately in solution phase by releasing the cyclic peptide from the bead. Substrate sequences of final hits with appropriate binding affinity are determined by partial Edman degradation-mass spectrometry (PED-MS) of the corresponding linear peptide (Thakkar et al., 2006). Compared with other screening methods such as phage display and individually-synthesized-peptide-library based drug discovery, OBOC library screening provides a delicate platform for the rapid selection of diverse peptides, incorporating NAAs, and various chemical modifications including backbone cyclization.

Several bioactive macrocyclic peptides have been identified with the OBOC screening method, including human prolactin receptor (hPRLr) antagonist (Liu et al., 2009), calcineurin (Cn)/nuclear factor of activated T cell (NFAT) interaction inhibitor (Liu et al., 2011), HIV-1 capsid (CA)-human lysyl-tRNAsynthetase (hLysRS) interaction inhibitor (Dewan et al., 2012), and Ras-effector interaction inhibitor (Wu et al., 2013; Upadhyaya et al., 2015) (Table S1). hPRLr is involved in normal lactation and reproduction; however, an excessive hPRLr level can cause various reproductive disorders. A macro octapeptide library containing five continuous random amino acids (with 12 proteinogenic and 14 nonproteinogenic residues at each position) was screened against hPRLr, revealing two peptide hits with dissociation constant (*K*_D_) values of 2.9 and 2.0 μM. However, both the hits were shown to be binding to the hPRLr surface rather than the prolactin-binding site (Liu et al., 2009). In 2011, a macrocyclic inhibitor of Cn-NFAT protein–protein interaction was discovered by screening a macrocyclic OBOC library with seven random residues. The best resulting hit possessed *K*_*D*_ against Cn of 0.74 μM. Compared with the well-established immunosuppressor Cyclosporin A and FK506, the selected macrocyclic inhibitor bears a Val-Ile-Val-Ile-Thr sequence that specifically binds to the NFAT-binding site of Cn, therefore avoiding undesired inhibition of Cn phosphatase activity, which is triggered by Cyclosporin A and FK506 treatment (Liu et al., 2011). Another OBOC screening published in 2012 against HIV-1 CA led to a binder with ~500 nM *K*_D_ and *in vitro* inhibition of LysRS-CA interaction with an IC_50_ of ~1 μM. By inhibiting LysRS-CA interaction, the HIV virion loses its capability of selectively packaging primer tRNA^Lys^, thereby impeding virus proliferation (Dewan et al., 2012). In 2013, a selective macrocyclic inhibitor of K-Ras-effector interaction was reported with OBOC screening, bearing an *in vitro* inhibitory IC_50_ 0.7 μM, whereas IC_50_ of the best previously reported inhibitor is 7 μM (Wu et al., 2013).

## Protein Splicing Method

Besides the above-described chemical methods of synthesizing and screening backbone macrocyclic peptides, the same can be accomplished intracellularly using microbiological techniques. The intracellular environment confers the unique availability of the activity-based two-hybrid method of selection rather than affinity-based selection methods (Di Lallo et al., 2001), thus drastically increasing the incidence of bioactive hits in the selection process (Tavassoli, 2017).

**Figure 7:**
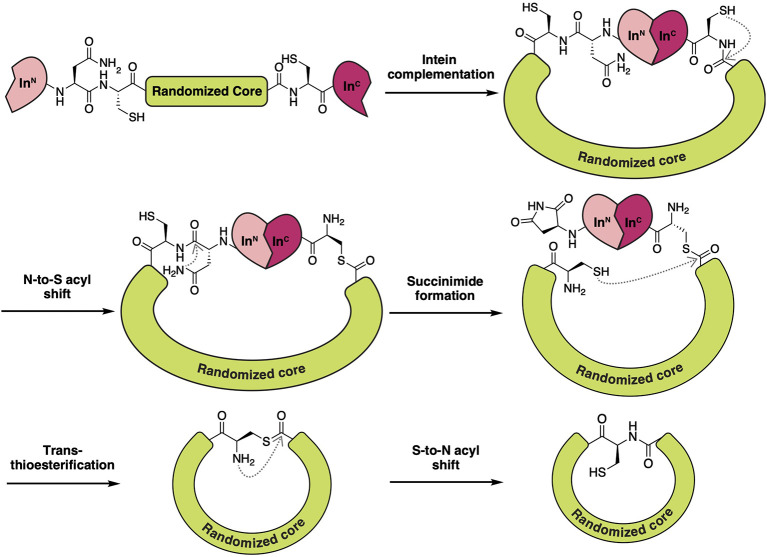
Schematic overview of the molecular processes involved in SICLOPPS to form backbone macrocyclic peptides. Through the utilization of a split-intein system, a macrocyclic intein linked peptide is formed upon complementation of both intein fragments. Intramolecular rearrangements such as N-to-S acyl transfer and asparagine succinimide formation open the opportunity for the now N-terminal Cys to restore the macrocycle by attacking the thioester link on the other side of the randomized region. This eliminates the succinimide tailed intein fragment and leaves, after trans-thioesterification, a Cys linked, genetically encoded backbone macrocyclic peptide that can be used for intercellular screening endeavors.

**Figure 8:**
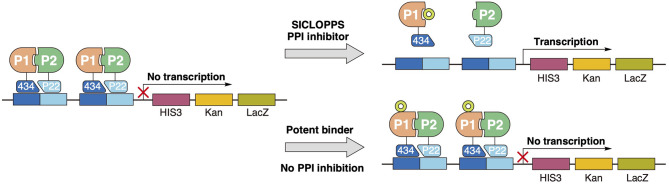
Peptide selection through reverse two-hybrid screening. Complementation of the transcription repressor domains 434 and P22, conjugated to the interaction proteins of interest, blocks the transcription of essential survival genes downstream. Only functional disruption of the target PPI interaction can restore transcription of the essential downstream genes and form a viable cell line. In contrast to other conventional screening methodologies, potent binding to the target will not constitute a “hit” unless this binding can functionally inhibit the targeted interaction.

Theoretically, Reverse Two-Hybrid (R2H) screening (Figure 8) would pair well with any high-throughput method of producing candidate inhibitors *in vivo*. For the discovery of backbone macrocyclic peptides, however, it is commonly paired with the split-intein circular ligation of peptides and proteins (SICLOPPS) methodology (Tavassoli, 2017; Valentine and Tavassoli, 2018). SICLOPPS utilizes the natural process of intein spicing to generate macrocyclic peptides (Figure 7). By placing a randomized sequence between a C-terminal and N-terminal intein domain, a backbone macrocyclic peptide library will be generated as a byproduct of the intein splicing process (Scott et al., 1999). Being a fully genetically encoded screening system, SICLOPPS is simple and accessible to labs with basic microbiological capabilities (Tavassoli and Benkovic, 2007).

In addition, SILOPPS is flexible for use with various expression hosts. While *Escherichia coli* is the preferred host organism for intein-mediated backbone macrocyclic peptide expression, the process has been successfully modified to adapt yeast display (Barreto et al., 2009; Kritzer et al., 2009; Bharathikumar et al., 2013; Valentine and Tavassoli, 2018), and human B cell display (Kinsella et al., 2002). Furthermore, the intein sequences that are used to generate backbone macrocyclic peptides are also consistently being improved to yield faster (Townend and Tavassoli, 2016) and remote controllable (Di Ventura and Mootz, 2019) splicing reactions. SICLOPPS' greatest strength, its plasmid-encoded nature, which makes it simple and accessible, also confers to the system its greatest weaknesses. Library sizes are limited not to the theoretical diversity of the peptides but to the physical transfection limit of the host cells (Valentine and Tavassoli, 2018), which, at its highest in *E. coli*, is roughly 10^9^ species (Dower et al., 1988). Although the system is capable of incorporating a single additional NAA through stop codon suppression, it is mostly limited to the 20 PAAs (Young et al., 2011). In addition, due to being spliced in macrocyclic form, post-translational modifications are also unavailable. These weaknesses are compensated by, arguably, the greatest boon the intracellular environment offers: activity-based two-hybrid screening.

Two-hybrid screening is a simple and popular genetic approach for analyzing and mapping PPIs within the cellular environment (Young, 1998; Mehla et al., 2017). By coupling proteins of interest to a split transcription factor that modulates survival-essential genes, only strains with interacting proteins are able to survive and be analyzed. Logically, by inverting the polarity of the screen, R2H (Figure 8), wherein complementation of the transcription factors will stop cell growth is, compared to affinity-based screens used by *in vitro* systems, a vastly superior screening platform for functional PPI inhibitors (Leanna and Hannink, 1996; Barr et al., 2004).

In combination, SICLOPPS and R2H have been able to discover novel bioactive peptides against difficult to target yet very worthwhile PPIs, such as the interaction between *Bacillus antharacis* protective antigen and the human CMG2 receptor that is essential for the cellular uptake of anthrax lethal and edema toxins with a *K*_*D*_ of 38.2 μM (Male et al., 2017). They have also enabled the development of cell-permeable inhibitors of the HIV Gag protein interaction with human TSG101, essential for HIV virus budding with a *K*_*D*_ of 11.9 μM (Lennard et al., 2019). The system can also be utilized to discover inhibitors of protein dimerization like the cyclic peptide inhibitors found to prevent the homodimerization of IDOL E3 ubiquitin ligase with a *K*_*D*_ of 4.6 μM (Leitch et al., 2018) and BCL6 with a *K*_*D*_ of 142 μM (Osher et al., 2018). In addition, even correctors of protein misfolding can be identified using SICLOPPS-based screening (Matis et al., 2017).

All in all, SICLOPPS combined with R2H screening provides a simple and accessible platform for function-based discovery of canonical backbone macrocyclic peptides with affinities ranging in the low to moderate μM scale (Table S1). Unlike other discovery platforms, however, it is constrained to the tolerance of its host cell, and drastic modifications, improvements, or expansions to the currently established system would be very challenging to develop.

## *In vitro* Synthesis of Backbone Macrocyclic Peptides

This section discusses an *in vitro* approach using genetic code reprogramming to express backbone macrocyclic peptides without relying on peptide ligation enzymes. Because of the uniqueness of this approach, this section includes an introduction of the technological history, followed by the application to the expression of backbone macrocyclic peptides.

### Genetic Code Manipulation Technologies for Incorporation of NAAs

**Figure 9:**
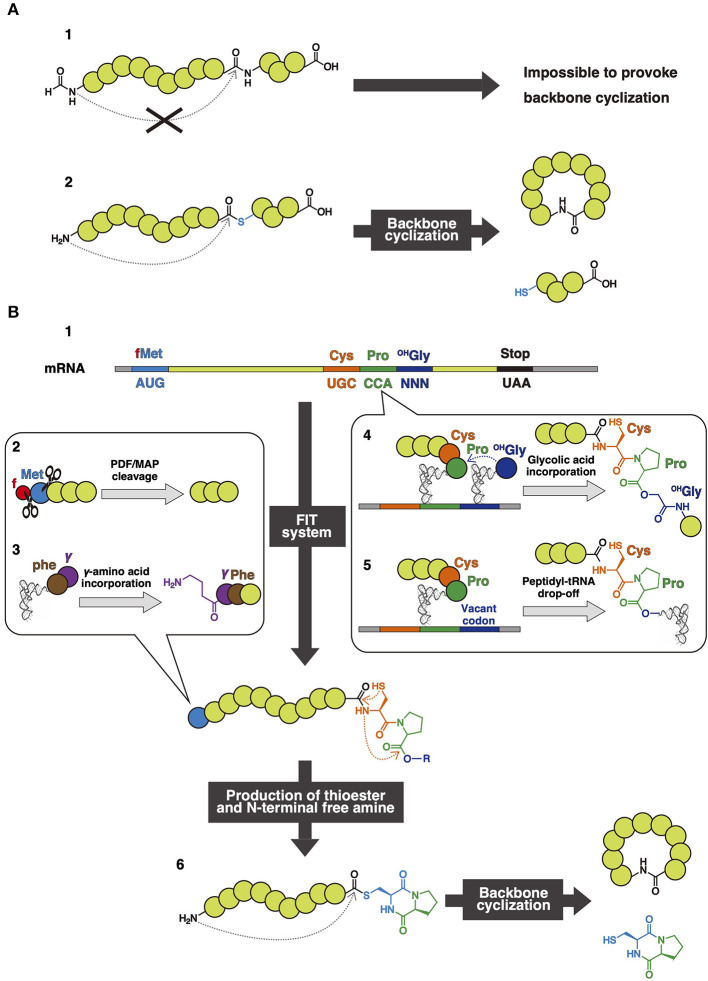
**(A)** General thioester activated backbone cyclization. A general translated peptide (1). A peptide containing N-terminal free amine and thioester to provoke backbone cyclization (2). **(B)** Dkp-thioester activated backbone cyclization. An mRNA sequence to perform dkp-thioester activated backbone cyclization (1). PDF/MAP cleavage for the production of free amine (2). Initiation codon reprogramming for expression of dipeptides containing γ-amino acid (3). The generation of an ester bond in the nascent peptide chain with expression of Cys, Pro, and ^OH^Gly (4), or Peptidyl-tRNA drop-off (5). Dkp-thioester activated backbone cyclization (6).

In nature, the ribosome is able to synthesize linear peptides consisting of 20 PAAs based on mRNA sequences. The *E. coli* ribosomal translation system has been reconstituted *in vitro* with purified translation components (PURE system) (Shimizu et al., 2001). One of the advantages of such reconstituted translation systems is the rapid and accurate polymerization of amino acids (40 amino acids/sec), which is significantly faster than that of chemical synthesis. In addition, these systems enable the facile construction of a peptide library with high diversity from the corresponding mRNA library and can be coupled with high-throughput screening methodologies, such as mRNA display (Nemoto et al., 1997). Unfortunately, the ribosomal translation system is intrinsically unable to synthesize backbone macrocyclic peptides because the translated peptides are initiated with formyl methionine (fMet), and amide bonds in the backbone have insufficient reactivity to provoke backbone cyclization (Figure 9A-1). The backbone cyclization of a peptide could be achieved by exposing the N-terminal free amine and activating the terminal acyl residue in the backbone. Figure 9A-2 shows a peptide containing a free amine at the N-terminus and exemplifies the process of acyl and thioester activation that is used for backbone cyclization of peptides in organic synthesis (Zheng et al., 2012; Thapa et al., 2014; Thell et al., 2016). The N-terminal free amine attacks the activated acyl residue to provoke a nucleophilic substitution reaction, leading to eventual backbone cyclization. N-terminal free amine can be produced relatively easily through enzymatic methods (Kawakami et al., 2009). However, to introduce such activated functional groups into peptide backbone through ribosomal synthesis, methodologies to manipulate the genetic code to allow the incorporation of NAAs are needed.

Genetic code manipulation technologies, such as stop codon suppression, programmed frame-shift suppression, and genetic code reprogramming, make it possible to solve this issue. Amber suppression is one of the stop codon suppression methods, wherein orthogonal tRNA_CUA_ charged with an NAA is assigned to the amber stop codon, producing peptides containing the NAA (Noren et al., 1989; Wang et al., 2001). However, the competition of NAA-tRNA with release factor 1 (RF1) limits the available amino acids to those with high incorporation efficiencies. This restriction can be overcome by removing RF1 from the translation mix (Wang et al., 2009; Johnson et al., 2011). However, only two of the three stop codons are at any time available for suppression, and thus, only two types of NAAs can be incorporated into a peptide.

The programmed frame-shift suppression method utilizes four-base codons and amber suppression together, making it possible to incorporate more than three NAAs at the same time (Magliery et al., 2001). The design of four-base codons generally relies on rare codons, the codons observed at low frequency in mRNAs of a particular organism. For example, the rare Arg AGG codon in the *E. coli* translation system is used for the design of a four-base codon, AGGA, and NAA-tRNA_UCCU_ is assigned to AGGA, producing a peptide containing the NAA. However, Arg-tRNA_CCU_ or Arg-tRNA_mnm5UCU_ (mnm^5^U, 5-methylaminomethyluridine) competes with AGGA, resulting in misincorporation of Arg and causing a frame-shift to appear. This misincorporation also significantly lowers the expression level of the designed peptide (Ohtsuki et al., 2005).

The genetic code reprogramming method replaces PAAs with NAAs to incorporate multiple NAAs. This method requires the preparation of vacant codons, to which NAA-tRNAs are assigned. To prepare a vacant codon, the PAA, and the corresponding aminoacyl-tRNA synthetase (ARS) are removed from the reconstituted translation system. The disadvantage of this method is that no more than 20 amino acids can be utilized at the same time due to the sacrificing of PAA for NAA incorporation. However, Iwane et al. recently reported that this can be overcome through artificial division of four-codon boxes into two. For instance, the GUN codon in Val's codon box was divided into GUC and GUG, to which *N*-methyltyrosine and Val were assigned, respectively. Therefore, Val was not sacrificed for NAA incorporation (Iwane et al., 2016). They demonstrated this through the expression of a model peptide containing 23 different amino acids (20 PAA + 3 NAA).

The NAA-tRNAs required for the genetic code manipulation can be synthesized by chemical or enzymatic methods (Bain et al., 1992; Wang et al., 2001). For instance, an aminoacylation ribozyme, flexizyme, is able to catalyze aminoacylation of a tRNA with an amino acid bearing a specific leaving group. Owing to the broad tolerance of flexizyme for tRNAs and amino acids, including NAAs, various NAA-tRNAs can be prepared. The combination of a reconstituted *E. coli* translation system and NAA-tRNAs prepared by flexizyme is referred to as the flexible *in vitro* translation (FIT) system, which enables the synthesis of various peptides containing NAAs by means of genetic code reprogramming (Yamagishi et al., 2011; Katoh et al., 2017; Passioura et al., 2018a; Katoh and Suga, 2019).

### *In vitro* Thioester-Activated Backbone Cyclization

As previously mentioned, the thioester bond is vulnerable to nucleophilic attack compared to the amide bond because the thioester's conjugated system has lower stability, and thiolate is a good leaving group. Therefore, this group plays an important role in the ligation of peptides and proteins as well as backbone cyclization (Blanco-Canosa and Dawson, 2008; Zheng et al., 2012; Thell et al., 2016). To accomplish backbone cyclization, it is necessary to generate a peptide containing a thioester group in the backbone and an N-terminal free amino group. This amino group then attacks the thioester to accomplish backbone cyclization while the thiolate leaving group is separated (Figure 9A-2).

In 2007, Kawakami and Aimoto reported an aqueous-compatible synthetic method to produce a diketopiperazine (dkp)-thioester as an intermediate that subsequently generated a rearrangement of cysteinyl-prolyl ester (CPE) at the C-terminus (Kawakami and Aimoto, 2007). Inspired by this work, a FIT-based method for the translation of peptides with a C-terminal Cys-Pro-^OH^Gly (glycolic acid), equivalent to CPE, was devised (Kawakami et al., 2009) (Figure 9B-4). This resulting peptide undergoes rearrangement to form a C-terminal dkp-thioester. Production of dkp can also be induced by peptidyl-tRNA drop-off caused by the artificial stalling of the ribosome during the elongation step (Kang et al., 2011). The peptidyl-tRNA has a Cys-Pro region in the C-terminal connecting with the 3'-terminal hydroxyl group of tRNA through an ester bond that is chemically analogous to ^OH^Gly. Therefore, after the peptidyl-tRNA drop off, the dkp-thioester is produced by the same rearrangement chemistry (Figure 9B-5).

Because fMet serves as an initiator in bacterial translation systems, the formyl group must be cleaved off to expose the N-terminal amine in order to cyclize through amine-dkp substitution. Therefore, a custom FIT system to eliminate the initiator fMet was prepared by adding peptide deformylase (PDF), which cleaves the formyl group of fMet. Then, methionine aminopeptidase (MAP) cleaves the N-terminal Met, which enables the introduction of other arbitrary amino acids than Met at the N-terminus (Figure 9B-2). When Cys emerges in this initial position, NCL will be provoked, and thus the backbone cyclization will be accomplished faster than that of methionine (Kimura et al., 2006).

Using the aforementioned method, dkp-thioester activation and cleavage of fMet lead to spontaneous backbone cyclization after peptide translation (Figure 9B-6). Several backbone macrocyclic peptides such as epidemnamide, as well as bicyclic and tetracyclic peptides cross-linked with disulfide bonds, such as sunflower trypsin inhibitor (SFTI-1) and rhesus θ defensin-1 (RTD-1), have been synthesized in this way (Kawakami et al., 2009). When synthesizing bicyclic and tetracyclic peptides, Cys was placed next to fMet to accelerate the cyclization through NCL after the cleavage of methionine.

This cyclization method enables the incorporation of more NAAs into backbone macrocyclic peptides using the FIT system. In 2009, scleramide and RTD-1 variants were expressed (Kawakami et al., 2009). Both of these peptides contain *N-*methylated amino acid residues, which increase protease resistance (Chatterjee et al., 2008; Doedens et al., 2010). This RTD^Me^-1 variant containing three *N-*methylated amino acids, ^Me^Gly, ^Me^Ala, and ^Me^Phe, inhibits protease activity (IC_50_ = 4.0 μM) to the same extent as wild-type RTD-1 (IC_50_ = 2.7 μM) produced in this manner and composed of only PAAs. In addition, Cys-Pro-^OH^Gly cyclization coupled with initiator codon reprogramming of polypeptides (ranging from dipeptide to pentapeptides) allows for the expression of peptides containing d-α-amino acids as well as β-/γ-amino acids, which are intrinsically difficult to incorporate into a peptide through traditional chain-elongation methods (Ohshiro et al., 2011) (Figure 9B-3).

### Construction of a Backbone Macrocyclic Peptide Library and Its Screening

**Figure 10:**
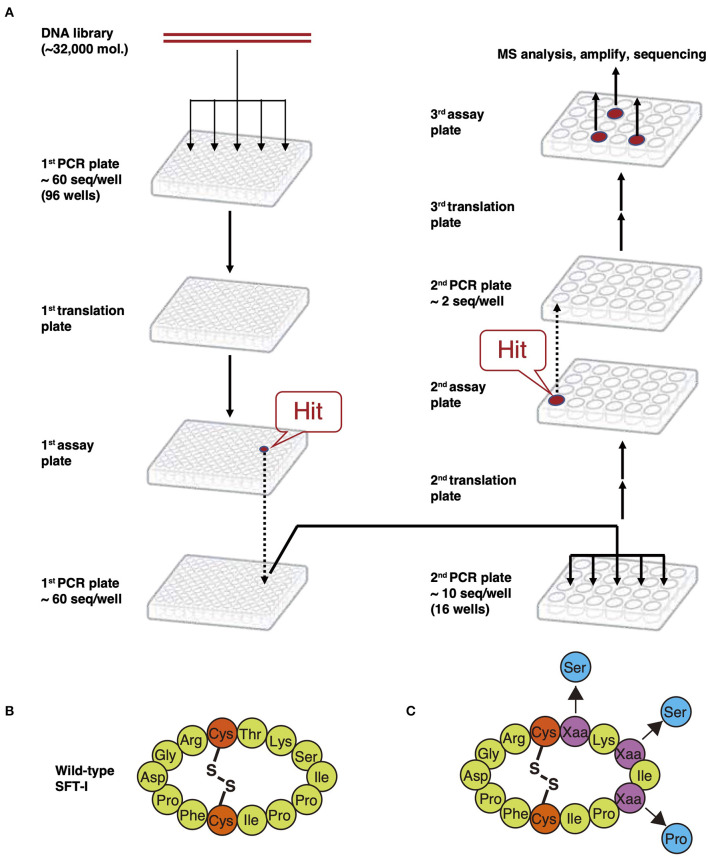
Limiting-dilution PCR deconvolution technology. **(A)** The procedures of limiting-dilution PCR. An mRNA library is transcribed from the corresponding DNA, which is separated into a 96-well plate in advance. Then, the mRNAs are translated to the peptides, and which are subsequently assayed separately in each well. The DNA in wells containing hit compounds is amplified by PCR to produce the next DNA library encoding more active peptides than the previous DNA library. This new library is then split into a new 96-well plate, and the cycle is repeated. The high-activity peptides converged after repeated cycles are identified by MS analysis and sequencing. **(B)** Structure of wild-type SFT-1. **(C)** Backbone macrocyclic peptide library based on SFT-1 sequence. Purple Xaa circles mean the randomized amino acids. Blue circles mean the mutation of amino acids on the obtained mutant SFT-1.

The FIT system-based cyclization method can be applied to the construction of peptide libraries with a theoretical diversity of ~10^13^ copies containing NAAs. Peptides are translated from a semi-randomized mRNA library, wherein the sequence between initiator AUG and the terminal UGC is randomized (Figure 9B-1). It can then be coupled with a screening system such as limiting-dilution PCR deconvolution technology (Figure 10A). In this methodology, an mRNA library is transcribed from its corresponding DNA library, which is split into a 96-well plate in advance. Then, the mRNAs are translated to peptides, and which are subsequently assayed separately in each well. Because DNA and the corresponding peptides coexist in the same well, the DNA in wells containing hit compounds is amplified by PCR to produce the next DNA library encoding more active peptides than previous DNA libraries. This new library is then split into a new 96-well plate, and the cycle is repeated. The hig- activity peptides converged after repeating cycles are identified by MS analysis and sequencing. In 2009, Kawakami et al. demonstrated the utility of this screening system by the discovery of SFT-1 mutant with an inhibitory activity equivalent to wild-type SFTI-1 (Kawakami et al., 2009). This wild-type peptide is a 14-mer backbone macrocyclic peptide containing only PAAs and a single internal disulfide bridge (Figure 10B). Based on this SFTI-I scaffold, a DNA library wherein three amino acids of the peptide were randomized by NNK codon was designed. Following translation, a backbone macrocyclic peptide library originating from ~60 unique compounds was obtained (Figure 10C). Finally, an SFTI-1 variant with an IC_50_ of 13.4 ± 0.7 nM was discovered, comparable to wild-type SFTI-1 (Figure 10C) (IC_50_ = 12.1 ± 0.3 nM). Nevertheless, this screening method is limited in that it can only be used to assay small libraries (<10^5^ copies) and thus cannot make full use of the capabilities of the merits of the FIT system, which is capable of synthesizing libraries containing up to ~10^13^ copies (Table S1).

### Backbone Cyclization Compatible With mRNA Display

**Figure 11:**
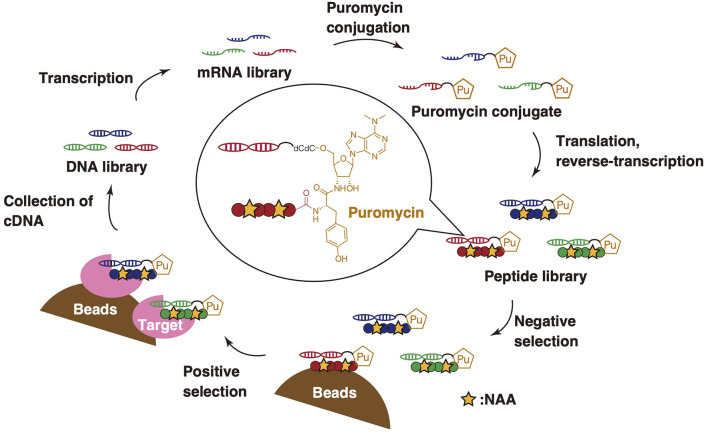
General scheme of the RaPID system. An mRNA library is transcribed from a DNA library and is then conjugated to puromycin. The peptides containing NAAs are subsequently translated based on mRNAs by employing genetic code reprogramming. At the end of translation, each puromycin combined with the mRNAs is connected to the C-terminus of corresponding peptides via an amide bond to produce a peptide-puromycin-mRNA library. Peptide ligands are obtained by affinity-based screening from its library through positive and negative selection. cDNAs from binding peptides are subsequently collected and amplified by PCR, and, finally, the cycle of the RaPID system is finished.

mRNA display is a reliable methodology for mass peptide library screening (~10^13^ members) and has been used for peptide drug-discovery (Josephson et al., 2014; Huang et al., 2019) (Figure 11). It is superior to other screening methodologies in terms of rapidness and facility to select peptides based on affinity potencies against proteins of interest. The combination of mRNA display and the FIT system is referred to as the random nonstandard peptides integrated discovery (RaPID) system, which enables the screening of peptides containing NAAs (Yamagishi et al., 2011).

In RaPID display, a randomized mRNA library is transcribed from a DNA library and conjugated to a puromycin linker. Then, peptides containing NAAs are translated by employing genetic code reprogramming and conjugated to the cognate mRNA template via the puromycin linker. Peptide ligands are obtained from the library by affinity-based screening. The selection will follow cycles of negative and positive selection to enrich potent, target-specific binding peptides. In “negative selection,” the peptide library is exposed to target-free magnetic beads in order to remove peptides binding to the beads, whereas “positive selection” involves collecting peptides binding to the target. Following these steps, cDNA from binding peptides is collected and amplified by PCR, finishing the cycle of the RaPID system. After several cycles of this screening, the peptide sequences are obtained by sequencing of the enriched cDNA attached to the peptide ligands with next-generation sequencing. Suga et al. have applied the RaPID system to screen thioether-closed macrocyclic peptide libraries (those cyclized via head-to-sidechain) to discover binders and has proven to yield potent inhibitors but also potent activators (Ito et al., 2015; Passioura et al., 2018b; Nitsche et al., 2019).

Unfortunately, mRNA display, including the RaPID system, intrinsically cannot be applied to the screening of backbone macrocyclic peptides due to the loss of the C-terminal peptide region linked to mRNA upon backbone cyclization. However, Takatsuji et al. have recently developed the synthesis of backbone macrocyclic peptide compatible with mRNA display, in which mRNA can be linked to the backbone macrocyclic peptide via a side chain linker (Takatsuji et al., 2019). This method consists of the following three steps: expression of a desired peptide by employing genetic code reprogramming (1st step), side chain-based coupling of the residues in the peptide that become the backbone cyclic peptide and the linker peptide whose C-terminal is connected to the corresponding mRNA/cDNA (2nd step), and then backbone cyclization (3rd step).

**Figure 12:**
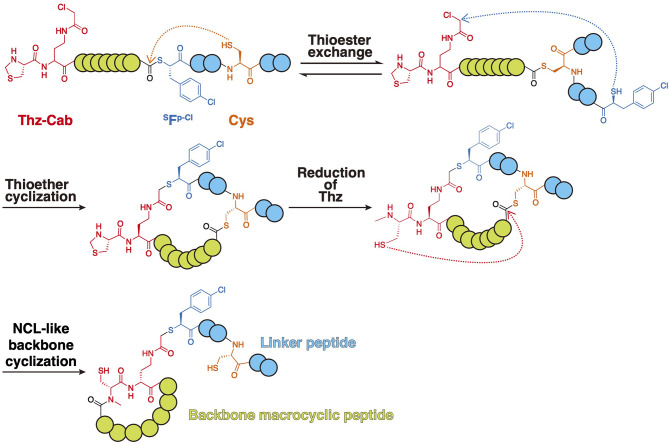
Synthesis of backbone macrocyclic peptides compatible with mRNA display. A linear peptide containing Thz-Cab, ^HS^F^p−Cl^, and subsequent Cys was transcribed by employing a genetic code reprogramming system. After spontaneous thioester exchange between the thioester and Cys, the resulting linear peptide spontaneously cyclized between the thiol Cab residue. Then, reduction of Thz by NaBH_3_CN produced *N-*methyl Cys, which provoked NCL-like backbone cyclization. Green circles show potential backbone macrocyclic peptide. Blue circles show linker peptide.

To accomplish this, a peptide containing (*R*)-thiazolidine-4-carboxylic acid (Thz), (*S*)-2-amino-4-(2-chloroacetamido) butanoic acid (Cab), thio acid bearing a *p*-chlorophenylalanine (^HS^F^p−Cl^), and Cys was expressed (1st step) (Figure 12). After spontaneous thioester exchange between the thioester and Cys, the resulting linear peptide spontaneously cyclized between the thiol Cab residue (2nd step). Then, reduction of Thz by NaBH_3_CN produced *N-*methyl Cys, which provoked NCL-like backbone cyclization (3rd step). This produced a backbone macrocyclic peptide that was still conjugated to its encoding puromycin-mRNA complex, allowing the RaPID display to be used for the screening of backbone macrocyclic peptides. This screening methodology will allow the rapid screening of backbone macrocyclic peptide ligands with a far bigger peptide library than previous screening methodologies.

## Conclusion and Perspective

Backbone macrocyclic peptides are an exceptionally powerful scaffold for drug discovery. With a size between those of small molecules and biological ligands, backbone macrocyclic peptides represent a golden middle ground, featuring potent target affinity, specificity, and potential cell permeability. Various chemical and biological approaches allow concise synthesis of diverse backbone macrocyclic peptides and their libraries that are compatible with high-throughput screening methods, resulting in promising peptide ligands against most biological targets. Here we looked at enzymatic or chemical synthetic approaches for backbone macrocyclic peptide libraries, as well as the library construction methodologies, such as the chemical approach of OBOC, the microbiological SICLOPPS, limiting-dilution PCR deconvolution, and the biochemical mRNA display methods and their screening. These screening methods make ultra-high-throughput screening accessible to nearly any lab intent on pursuing this, without a need for specialized facilities, dedicated molecule libraries, or intense automatization processes. The aforementioned peptide ligands are summarized in Table S1. Each of these methods boasts its own strengths and carries its own weaknesses. Because only SICLOPPS is compatible with activity-based R2H screening, it is more reliable in discovering bioactive hits, whereas the other methods are more prone to discover high-affinity binders that lack any biological activity. Nevertheless, these methods can utilize a multitude of NAAs within their library, giving more chemical space to discover bioactive peptides.

Nowadays, researchers are constantly developing the synthesis of peptides containing NAAs, aiming to add more functionalities, such as exceptional stability and cell-membrane permeability, or to form specific tertiary structures to discover even better peptide therapeutic agents (Groß et al., 2016; Goto and Suga, 2018). So, the chemical space of available peptides is expanding. On the other hand, some synthesis of backbone macrocyclic peptide-compatible screening technologies can be coupled with the introduction of peptides containing NAAs. Taking these synthetic methods and screening methods together, the bioactive peptide will be discovered from a library with a larger chemical space.

## Author Contributions

KS planned the framework for this article. KS, WL, and RN have written individual chapters and prepared figures. All authors contributed to the writing of this review.

## Conflict of Interest

The authors declare that the research was conducted in the absence of any commercial or financial relationships that could be construed as a potential conflict of interest.
