# Supplementary material for: Methodologies for Backbone Macrocyclic Peptide Synthesis Compatible With Screening Technologies
Source: Front Chem. 2020 Jun 18;8:447. doi: 10.3389/fchem.2020.00447 (PMC7314982; doi:10.3389/fchem.2020.00447)
Supplement: Supplementary file 1 [file Table_1.pdf]

Screening method	Library size	NAA	Peptide	Target	Binding affinity	Inhibition/EC_{50}	Cell assay	Reference
OBOC	$<10^7$	✓	cyclo(ARYQSRVE)	Human prolactin receptor (hPRLr)	2.0 μM	No activity	No	Liu et al., 2009
			cyclo(RR^{D}LIR-Pip-PVIVITAAE)	Calcineurin (Cn)/nuclear factor of activated T cell (NFAT) interaction	0.74 μM	N/A	No	Liu et al., 2011
			cyclo(^{D}Al-Fpa-RYW^{D}A^{D}AE)L	HIV-1 capsid (CA)/human lysyl-tRNAsynthetase (hLysRS) interaction	1.1 μM	0.61 μM	No	Dewan et al., 2012
			cyclo(RR-^{D}Nal-R-Fpa-^{D}Nle-Dkb-Pip-^{D}homoPheE)	K-Ras/Raf interaction	0.83 μM	0.7 μM	Yes	Wu et al., 2013; Upadhyaya et al., 2015
SICLOPPS-R2H	$<10^9$	A single additional NAA	CLR(4-Cl-F)T	PA – CMG2	14 μM	N/A	No	Male et al., 2017
			KRL74	HIV GAG P6 – UEV	11.9 μM	5.44 μM	Yes	Lennard et al., 2019
			Cyclo-CF-Cha-LYT	IDOL E3 – IDOL E3	4.6 μM	20 μM	Yes	Leitch et al., 2018
			cyclo-CIYYCV	BCL6 – BCL6	50 μM	N/A	No	Osher et al., 2018
Limiting-dilution PCR	$<10^5$	✓	Cyclo-(CSKSIPICFPDGR)	Trypsin	N/A	13.4±0.7 nM (IC_{50})	No	Kawakami et al., 2009
mRNA display	$<10^{13}$	✓	-	-	-	-	-	-

Supplementary Table 1. Comparison of screening methodologies for backbone macrocyclic peptides.
